# Genome-wide expression profiling reveals increased stability and mitochondrial energy metabolism of the human liver cell line HepaRG-CAR

**DOI:** 10.1007/s10616-020-00384-w

**Published:** 2020-03-04

**Authors:** Aziza A. A. Adam, Aldo Jongejan, Perry D. Moerland, Vincent A. van der Mark, Ronald P. Oude Elferink, Robert A. F. M. Chamuleau, Ruurdtje Hoekstra

**Affiliations:** 1 Tytgat Institute for Liver and Intestinal Research, AG&M, Amsterdam UMC, University of Amsterdam, Meibergdreef 69-71, 1105 BK Amsterdam, The Netherlands; 2 Department of Clinical Epidemiology, Biostatistics and Bioinformatics, Amsterdam UMC, University of Amsterdam, Meibergdreef 9, 1105 AZ Amsterdam, The Netherlands; 3 Surgical Laboratory, Amsterdam UMC, University of Amsterdam, Meibergdreef 9, 1105 AZ Amsterdam, The Netherlands

**Keywords:** Constitutive androstane receptor, Dedifferentiation, Mitochondrial energy metabolism, Oxidative stress

## Abstract

Human liver cell line HepaRG is a well-known source of human hepatocyte-like cells which, however, displays limited biotransformation and a tendency to transform after 20 passages. The new HepaRG-CAR cell line overexpressing constitutive androstane receptor (CAR, NR1I3), a regulator of detoxification and energy metabolism outperforms the parental HepaRG cell line in various liver functions. To further characterize this cell line and assess its stability we compared HepaRG-CAR with HepaRG cells at different passages for their expression profile, ammonia and lactate metabolism, bile acid and reactive oxygen species (ROS) production. Transcriptomic profiling of HepaRG-CAR vs. HepaRG early-passage revealed downregulation of hypoxia, glycolysis and proliferation and upregulation of oxidative phosphorylation genesets. In addition CAR overexpression downregulated the mTORC1 signaling pathway, which, as mediator of proliferation and metabolic reprogramming, may play an important role in the establishment of the HepaRG-CAR phenotype. The ammonia and lactate metabolism and bile acid production of HepaRG-CAR cells was stable for 10 additional passages compared to HepaRG cells. Interestingly, bile acid production was 4.5-fold higher in HepaRG-CAR vs. HepaRG cells, whereas lactate and ROS production were 2.7- and 2.0-fold lower, respectively. Principal component analysis showed clustering of HepaRG-CAR (early- and late-passage) and HepaRG early-passage and not with HepaRG late-passage indicating that passaging exerted larger effect on the transcriptional profile of HepaRG than HepaRG-CAR cells. In conclusion, overexpression of CAR in HepaRG cells improves their bile acid production, mitochondrial energy metabolism, and stability, with the latter possibly due to reduced ROS production, resulting in an optimized source of human hepatocytes.

**Electronic supplementary material:**

The online version of this article (10.1007/s10616-020-00384-w) contains supplementary material, which is available to authorized users.

## Background

Highly differentiated human hepatocytes from proliferative sources are needed for hepatocyte-based in vitro models of human liver and for several clinical applications, such as Bio-artificial Livers (BALs). Yet, at present, hepatocytes deriving from different proliferative sources, as stem cells, induced pluripotent stem cells and liver cell lines, are lacking complex hepatic functions (van Wenum at al. 2014), and mature primary human hepatocytes (PHHs) are scarce and costly. The human liver cell line HepaRG displays a relatively high hepatic functionality and its transcriptome profile resembles that of PHHs most closely compared to other proliferative sources of human hepatocytes (Gao and Liu 2017; Kvist et al. 2018). During culturing, HepaRG cells progress in a period of 4 weeks from progenitor cells into a mixed population of hepatocyte-islands surrounded by cholangiocyte-like cells (Gripon et al. 2002). In our lab, we successfully applied the HepaRG cell line as a biocomponent of the AMC-BAL in an animal study (Nibourg et al. 2012). However, one of the major obstacles that limit the usage of HepaRG cells, is their tendency to transform after undergoing 20 passages from isolation with a split ratio of 1:5. The HepaRG cells then gradually lose their hepatic differentiation and acquire a mesenchymal phenotype with glycolysis-dependent energy metabolism, as evidenced by increased lactate production (Laurent et al. 2013; Adam et al. 2018a). This instability represents a hurdle towards the long-term and large-scale usage of HepaRG cells, as required for BAL application. In addition, the biotransformation properties, also deemed essential for BAL application, although higher compared to other proliferative sources of human hepatocytes, are, in part, still limited in absence of dimethylsulfoxide (DMSO) (Kvist et al. 2018; Andersson et al. 2012; Hoekstra et al. 2013). DMSO treatment increases biotransformation, but negatively affects cell viability and transcript levels of hepatic genes unrelated to biotransformation (Hoekstra et al. 2011). Moreover, for pharmacological applications, the HepaRG cells still prove insufficient in predicting liver-induced toxicity (Sison-Young et al. 2017; Bell et al. 2017). To improve biotransformation properties without DMSO treatment, we recently established a new stable cell line, HepaRG-CAR, by lentiviral overexpression of the constitutive androstane receptor (CAR, NR1I3) in HepaRG cells (van der Mark et al. 2017). CAR is a transcription factor involved in drug metabolism, but also in other processes, including energy metabolism, lipid homeostasis and cell proliferation (Yan et al. 2015). The resulting HepaRG-CAR cells exhibited increased biotransformation, including cytochrome P450 (CYP) activities and bilirubin conjugation. In addition, also albumin production, resistance to DMSO-induced toxicity, and NAD(P)H levels were elevated by CAR overexpression, however ammonia elimination remained unchanged (van der Mark et al. 2017).

To further investigate its potential as proliferative hepatocyte source we compared the transcriptional profile of the newly established HepaRG-CAR cell line and its parental cell line HepaRG using next generation RNA sequencing (RNA-seq) and assessed their stability upon serial passaging.

## Materials and methods

### Cell culture

HepaRG cells were kindly provided by Biopredic International (Rennes, France). The HepaRG-CAR line was developed by stable lentiviral overexpression of the nuclear receptor CAR, as described (van der Mark et al. 2017). This cell line is designated as HepaRG-CAR (RRID:CVCL_X149) in Cellosaurus (Bairoch 2018), and could be considered as a constituent of the human hepatocyte invitrome, which would be all the human liver cells expressing hepatocyte properties (Bols et al. 2017). HepaRG and HepaRG-CAR cells were maintained in William’s E-based culture medium (HepaRG medium) in a humidized atmosphere of 95% air and 5% CO_2_., as described (Gripon et al. 2002; Hoekstra et al. 2011), and the medium was refreshed twice/week. To test the stability of the cells upon serial passaging, the cultures were propagated starting at passage 15 (P15) for HepaRG and P19 for HepaRG-CAR from isolation. The cultures were passaged at a regular 1:5 ratio once per 2 weeks, and for every two passages (passage 15, 17, 19, 21, 23, 25, 27, 29 and 31) cells were seeded in parallel in 12-well culture plates for testing functionality and obtaining RNA after 4 weeks culturing.

### RNA isolation

PHHs were isolated from the healthy parenchyma in liver resection material from three caucasian patients, aged 40–68 (Table 1 for details), with no macroscopic signs of liver damage, by a modified 2-step collagenase perfusion technique, as described (Hoekstra et al. 2006). Cells were snap-frozen directly after isolation and kept in liquid nitrogen until RNA isolation. The procedure was in accordance with the ethical standards of the institutional committee on human experimentation (Medical Ethical Committee of the Academic Medical Center, University of Amsterdam, protocol number 03/024) and the Helsinki Declaration of 1975 and after obtaining written informed consent. Total RNA was isolated using the RNeasy kit (QIAGEN) from the three PHH isolates and 12 samples of fully differentiated cultures, including three samples from HepaRG early-passage cultures (P15 and P16), three HepaRG late-passage cultures (P21), three HepaRG-CAR early-passage cultures (P17) and three HepaRG-CAR late-passage cultures (P22), for more information refer to Table 1. The quality of the RNA was assessed by Agilent RNA 6000 Nano-Bioanalyzer. Samples with RNA integrity number above 7 were used for RNA-seq (all samples).

**Table 1:** Details of the isolated PHHs and monolayer cultures used for the RNA-seq analysis

Sample	Culture line	Passage	ID	Exonic reads
HepaRG	BB	P15	HepaRG early-passage, replicate 1	14,004,119
HepaRG	BG	P16	HepaRG early-passage, replicate 2	16,656,149
HepaRG	BG	P16	HepaRG early-passage, replicate 2	14,602,082
HepaRG	BB	P21	HepaRG late-passage, replicate 1	12,419,838
HepaRG	BB	P21	HepaRG late-passage, replicate 1	12,168,126
HepaRG	BG	P21	HepaRG late-passage, replicate 2	15,193,830
HepaRG-CAR	A	P17	HepaRG-CAR early-passage, replicate 1	12,122,156
HepaRG-CAR	A	P17	HepaRG-CAR early-passage, replicate 1	11,506,605
HepaRG-CAR	A	P17	HepaRG-CAR early-passage, replicate 1	11,285,599
HepaRG-CAR	A	P22	HepaRG-CAR late-passage, replicate 1 (excluded)	13,325,420
HepaRG-CAR	A	P22	HepaRG-CAR late-passage, replicate 1	13,483,596
HepaRG-CAR	C	P22	HepaRG-CAR late-passage, replicate 2	14,744,261
PHHs 1	NA	Fresh isolate	PHHs 1 (excluded). Donor: male, 68 years, liver metastasis	2,485,170
PHHs 2	NA	Fresh isolate	PHHs 2. Donor: female, 40 years, intraductular papilloma of the biliary tree	14,750,007
PHHs 3	NA	Fresh isolate	PHHs 3. Donor: female, 41 years, adenoma	8,037,487

In this table the culture line and passage number of HepaRG and HepaRG-CAR cultures are given, together with the new ID of the replicates and total number of exonic reads per sample (after filtering)

*NA* not applicable

### cDNA preparation and RNA-seq

A cDNA library was prepared from ribosomal-depleted RNA (50 ng input/sample) according to the Ovation^®^ RNA-Seq System V2 kit (Nugen) protocol. Next, the cDNA was fragmented, blunt ended, ligated to indexed (barcoded) adaptors and amplified with PCR using the Ovation^®^ Ultralow System V2 kit (Nugen) according to manufacturer’s protocol. Prior to RNA-seq analysis, the final library size distribution was determined using Agilent Bioanalyzer 2100. Fifteen cDNA libraries were prepared with one library per RNA sample. Next, all cDNA libraries were pooled and single-end sequenced (50 nucleotides) on two lanes of the Illumina HiSeq4000 platform.

### RNA sequencing data analysis

Raw sequencing data were subjected to quality control using FastQC and trimmed using Trimmomatic (v0.32). Reads were aligned to the human reference genome (hg38) using HISAT2 (v2.0.4). Gene level counts were obtained using HTSeq (v0.6.1) and the human GTF (gene transfer format) file from Ensembl (release 85). Samples from a different well, but from the same cell line, seeded from the same culture were considered to be technical replicates and their counts were summed, therefore (n = 1or 2)/group, refer to Online Resource 1. One of the PHHs sample was excluded, as it clearly exhibited a cancerous rather than hepatic transcriptional profile. Based on principal component analysis (PCA), one of the HepaRG-CAR late samples was identified as outlier and therefore excluded from downstream analysis. Statistical analyses were performed using the edgeR and limma R (v.3.4.1) and Bioconductor (v3.5) packages. Genes with more than one count in one or more samples were retained. The two most abundant genes (MT-RNR1 and MT-RNR2) were removed in order to stabilize the scaling factors. Count data were transformed to log2-counts per million (logCPM), normalized by calculating scaling factors using the trimmed mean of M-values method and precision weighted using voom. Differential expression was assessed using an empirical Bayes moderated t-test within limma’s linear model framework including the precision weights estimated by voom. Resulting *P-*values were corrected for multiple testing using the Benjamini–Hochberg false discovery rate (FDR). Additional gene annotation was retrieved from Ensembl (release 91) using the biomaRt R/Bioconductor package. PCA was performed on the logCPM values of the 5000 most variable genes (function prcomp). The variance explained by the first two principal components was calculated as percentage of the total variance. Geneset enrichment analysis was performed using CAMERA (limma package) with preset value of 0.01 for the inter-gene correlation using the Hallmark, C1, C2, C3, C5, C6 and C7 geneset collections retrieved from the Molecular Signatures Database (v6.0; Entrez Gene ID version. *P*-values were calculated for each geneset for two alternative hypotheses (‘up’ or ‘down’) and adjusted using the Benjamini-Hochberg FDR. Geneset variation analysis (GSVA) was performed using the GSVA package. Sample-specific geneset enrichment scores calculated by GSVA were clustered using Euclidean distance and complete linkage as agglomeration method (function hclust).

### Quantitative reverse transcription PCR (RT-qPCR)

Quantitative RT-PCR was performed as previously described (Hoekstra et al. 2005; Nibourg et al. 2013). Transcript levels were normalized for 18S ribosomal RNA and expressed as a % of the average of two human liver samples. Primer sequences and amplicon sizes are given in Table 2.

**Table 2:** Primers used in PCR analyses and amplicon size

Gene	Sense sequence	Anti-sense sequence	Size bp
*18S rRNA*	TTCGGAACTGAGGCCATGAT	CGAACCTCCGACTTTCGTTCT	151
*CAT*	TGGGATCTCGTTGGAAATAACAC	TCAGGACGTAGGCTCCAGAAG	146
*SOD1*	GGTGGGCCAAAGGATGAAGAG	CCACAAGCCAAACGACTTCC	227
*SOD2*	TTTCAATAAGGAACGGGGACAC	GTGCTCCCACACATCAATCC	109

### Hepatic function test

HepaRG and HepaRG-CAR fully-differentiated monolayer cultures of different passages were tested in 12-well plates (Corning) for their functionality, as described (Chiang 2009). Briefly, cultures were exposed to 1.5 mL test medium based on HepaRG medium supplemented with 1 mM carbamoyl glutamate, the allosteric activator of the urea cycle enzyme carbamoyl phosphate synthase 1, 1.5 mM NH_4_Cl, 2.27 mM d-galactose, 2 mM l-lactate and 125 µM testosterone (all compounds from Sigma Aldrich). During the function test, medium samples were taken at 0.75 h (0.5 mL) and at 24 h in which l-lactate, ammonia and bile acids were measured, as described (Adam et al. 2018a). At termination, the cells were washed with phosphate buffered saline and total protein/well was determined, as described (Adam et al. 2018a). Metabolic activities were calculated on basis of the changes in concentration in medium in time, normalized to the protein content per well (function test raw-data is given in Online Resource 1).

### Mitochondrial superoxide detection (MitoSOX)

MitoSOX based flow cytometric assay was used to detect mitochondrial superoxide in 4-week cultures of low-passage HepaRG and HepaRG-CAR cells in 24-well plates. MitoSOX targets to mitochondria, where oxidation by superoxide results into red fluorescence (Zhou et al. 2011). The cells were incubated with 250 µL of Hanks' balanced salt solution (Gibco) containing 10 mM HEPES (pH7.4, Sigma) and 5 µM freshly prepared MitoSOX (Thermo Fisher) for 0.5 h. Subsequently, the cells were trypsinized and fluorescence was analyzed by flow cytometry, data of MitoSox analysis is shown in Online Resource 1.

#### Statistical analysis

We performed one-way ANOVA test with Dunnett's post hoc test to compare the mean of the baseline passage of either HepaRG (P15) or HepaRG-CAR (P19) with the mean of every other passage analyzed within the same cell line. Furthermore, Student’s *t*-test was applied to compare the corresponding passages (P19–P25) in HepaRG vs. HepaRG-CAR. Analyses were performed with Prism version 7 (GraphPad Prism Inc). Data are expressed as mean ± SD; *P*-value < 0.05 was considered as significant.

## Results

### HepaRG-CAR cell line exhibits more stable hepatic functionality upon passaging

To compare the stability of the HepaRG and HepaRG-CAR cell lines, the cultures were passaged every 2 weeks, and their hepatic functionality was assessed once every two passages.

**Fig. 1:**
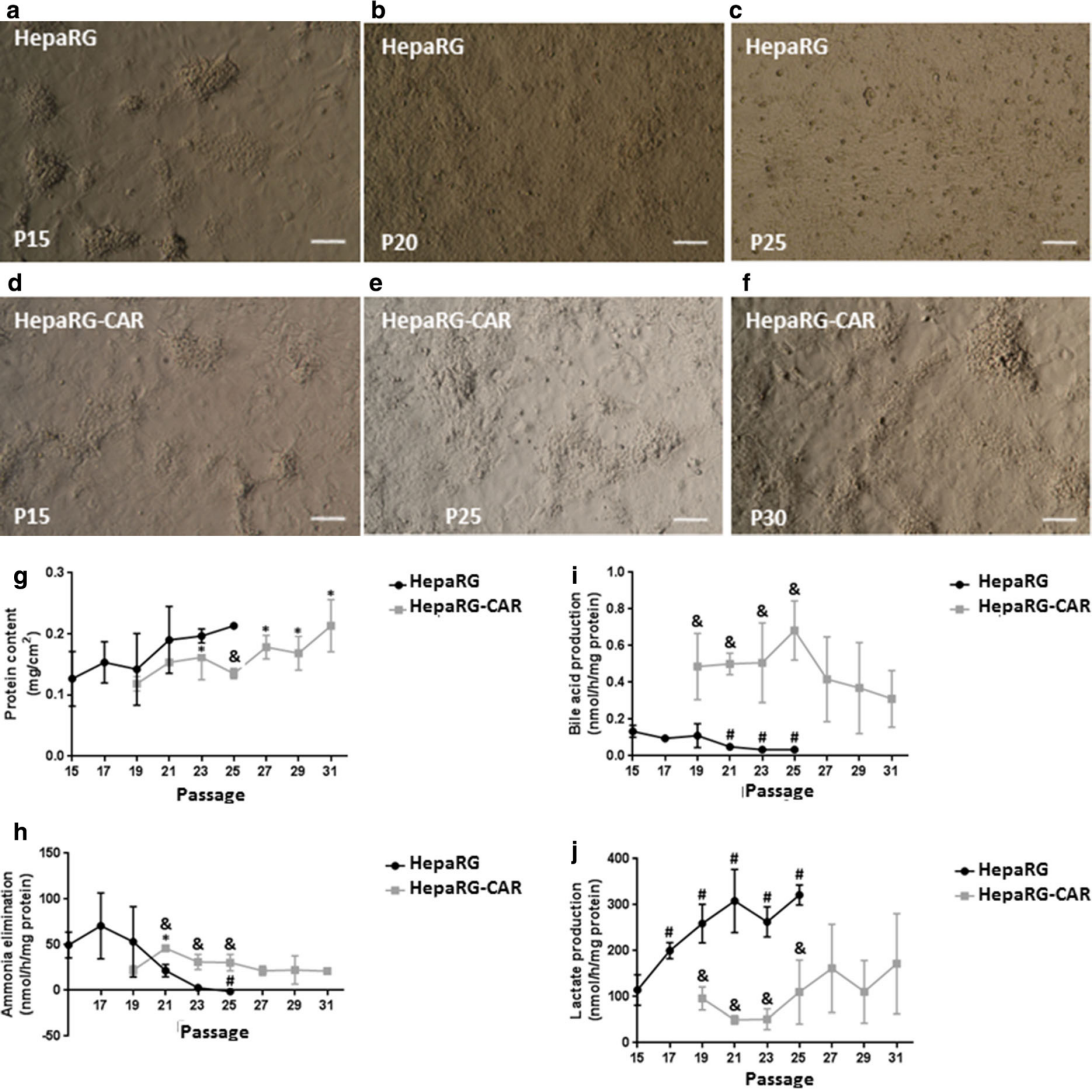
HepaRG-CAR morphology and functions are more stable upon passaging. **a**–**f** Morphology. Scale bar = 100 µM. **g** Protein content/well. **h** Ammonia elimination. **i** Lactate production. **j** Bile acid production, (n = 3–9/ 1–3 independent experiments). Significance is indicated by ^#^vs. HepaRG P15, *vs. HepaRG-CAR P19 and ^&^vs. HepaRG at the same passage

HepaRG cultures showed gradual morphological changes over passaging. At early passage (P15) the cultures displayed well-delineated hepatocyte-islands surrounded by flat cholangiocyte-like cells (Fig. 1a). The island structure was partially maintained after five passages (P20) (Fig. 1b), and was totally disrupted at P25 (Fig. 1c), and the cell size was reduced. In contrast, the island structure of HepaRG-CAR cultures showed no evident changes up to P30 (Fig. 1d–f), indicating increased morphological stability upon serial passaging at least up to 10 passages above the critical passage P20 of HepaRG cells.

Furthermore, we compared functionality and total protein content of HepaRG and HepaRG-CAR monolayers upon passaging, refer to Online Resource 1. Total protein content/well exhibited an increasing trend in both cell lines upon passaging (Fig. 1g). At P25, the total protein content was 1.6-fold higher in HepaRG vs. HepaRG-CAR cells. At late passages (P27–P31), the total protein content/well was significantly increased in the HepaRG-CAR line compared to baseline level. Ammonia elimination, which is a hepatocyte hallmark function, severely deteriorated with the passaging of HepaRG cells and converted into marginal ammonia production at P25 (Fig. 1h). In contrast, ammonia elimination by HepaRG-CAR cells displayed more stable profile through all tested passages, except for P21 which was 2.1-fold improved vs. baseline (P19) level. Notably, ammonia elimination by HepaRG-CAR cells was exceeding the level of HepaRG cells starting from P21. Similarly, bile acid synthesis, another essential hepatocyte function, declined 3.9-fold during the passaging of HepaRG cells from P15 until P25 (Fig. 1i) and was stable in HepaRG-CAR cells. In addition, bile acid synthesis was 4.5-fold higher in HepaRG-CAR cells vs. HepaRG cells at P19. For comparison, the average ammonia elimination and bile acid production of the HepaRG-CAR cells at P19-P31 were 29% and 31% the levels found in PHHs (Hoekstra et al. 2011, 2013). Lactate production, as a measure of mitochondrial dysfunction, raised gradually in HepaRG cells up to 2.8-fold difference at P25 vs. P15 (Fig. 1j). Again, HepaRG-CAR cells exhibited a stable lactate production during passaging. At P19 the lactate production in HepaRG-CAR cells was 2.7-fold lower when compared to P19 HepaRG cultures, suggesting that HepaRG-CAR cells possess an enhanced and sustainable mitochondrial function, although still not at the level of PHHs that actually eliminate lactate.

Collectively, these results show that HepaRG cultures lose their hepatic functionality upon passaging whereas HepaRG-CAR cells display a stable phenotype for 10 passages above the critical P20 in the parental cells and also show improved basal bile acid production and lactate metabolism, whereas ammonia clearance was comparable to HepaRG cells.

### The effect of passaging on the transcriptional profile of HepaRG-CAR cells is limited

**Fig. 2:**
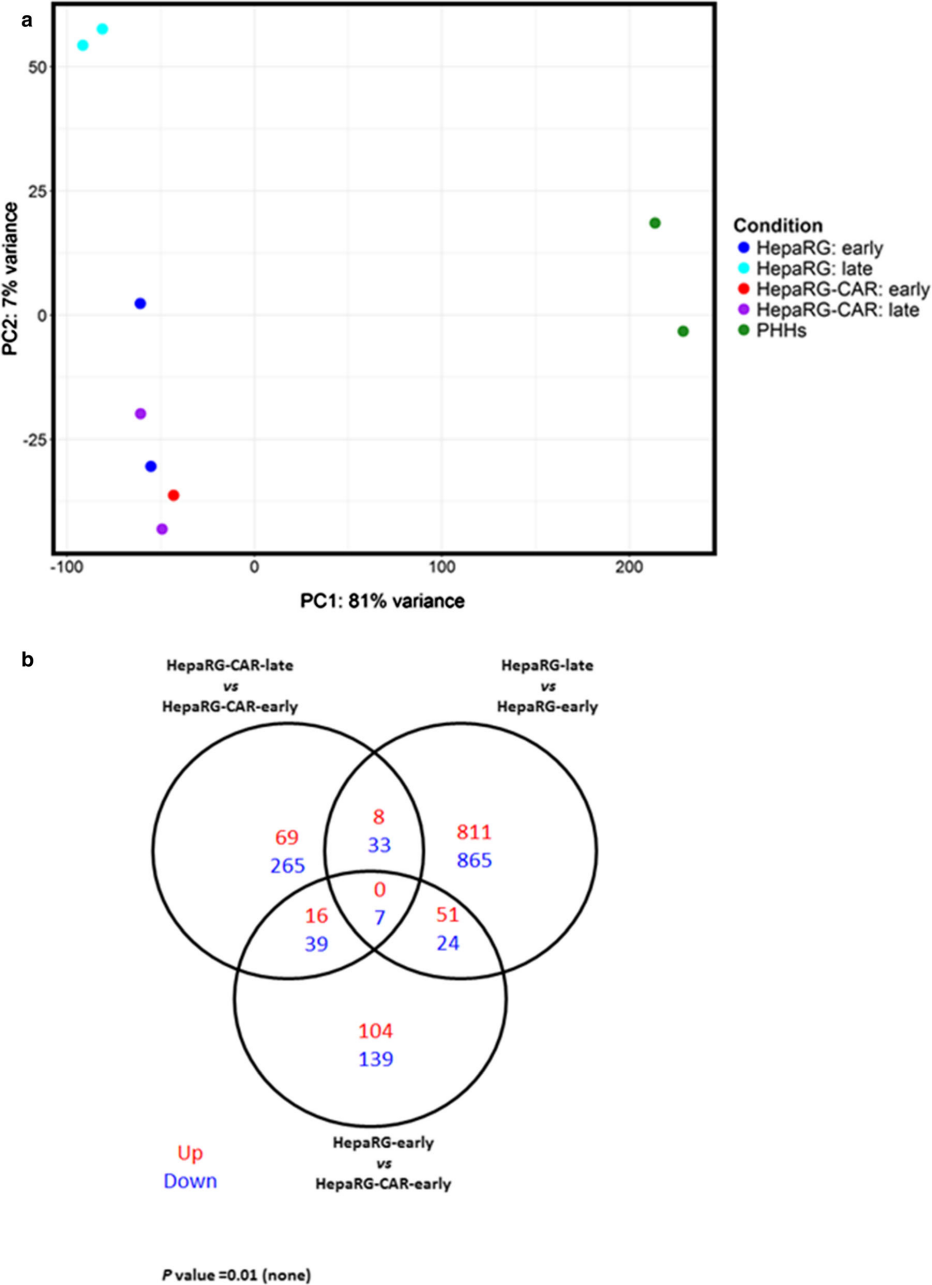
The effect of passaging on the transcriptome of the HepaRG-CAR cell line is limited. **a** Principal component analysis of gene expression data. Each symbol represents an individual sample. PC1 and PC2 indicate principal components 1 and 2. **b** Venn diagram of DEG, cut-off *P*-value 0.01 (not adjusted). Numbers shown in the center of the Venn diagram represent the significantly upregulated (up) and downregulated (down) DEG. The total number of up- and downregulated genes included in the analysis is depicted in the lower right corner. (Color figure online)

To investigate the molecular background of the increased hepatic differentiation and stability of HepaRG-CAR cells, RNA-seq was performed of the HepaRG and HepaRG-CAR fully differentiated cultures at different passages (early, i.e. P15–P17 vs. late, i.e. P21–P22) and their gene expression profiles were compared with those of two PHH isolates that served as gold standard. PCA revealed that the expression profiles of HepaRG and HepaRG-CAR cells, regardless of the passage number, were different from those of PHHs (Fig. 2a). Interestingly, expression profiles of HepaRG-CAR (early- and late-passages) cells were similar and clustered to some extent with those of the HepaRG early-passage cells, and deviated considerably from those of HepaRG late-passage cells (Fig. 2a).

Next, we identified differentially expressed genes (DEGs) between HepaRG and HepaRG-CAR cells. There were 171 upregulated genes and 209 downregulated genes between HepaRG vs. HepaRG-CAR at early-passage (Fig. 2b). Analysis of DEGs as a measure of the passaging effect on gene expression, revealed far less changes in HepaRG-CAR late- vs. early-passage with only 93 upregulated and 344 downregulated genes, compared to HepaRG late- vs. early-passage with 870 and 929 genes up- and downregulated respectively (Fig. 2b).

### Upon passaging, the transcriptional profile of HepaRG cells shifts towards dedifferentiation and cell proliferation with enhanced hypoxia

**Fig. 3:**
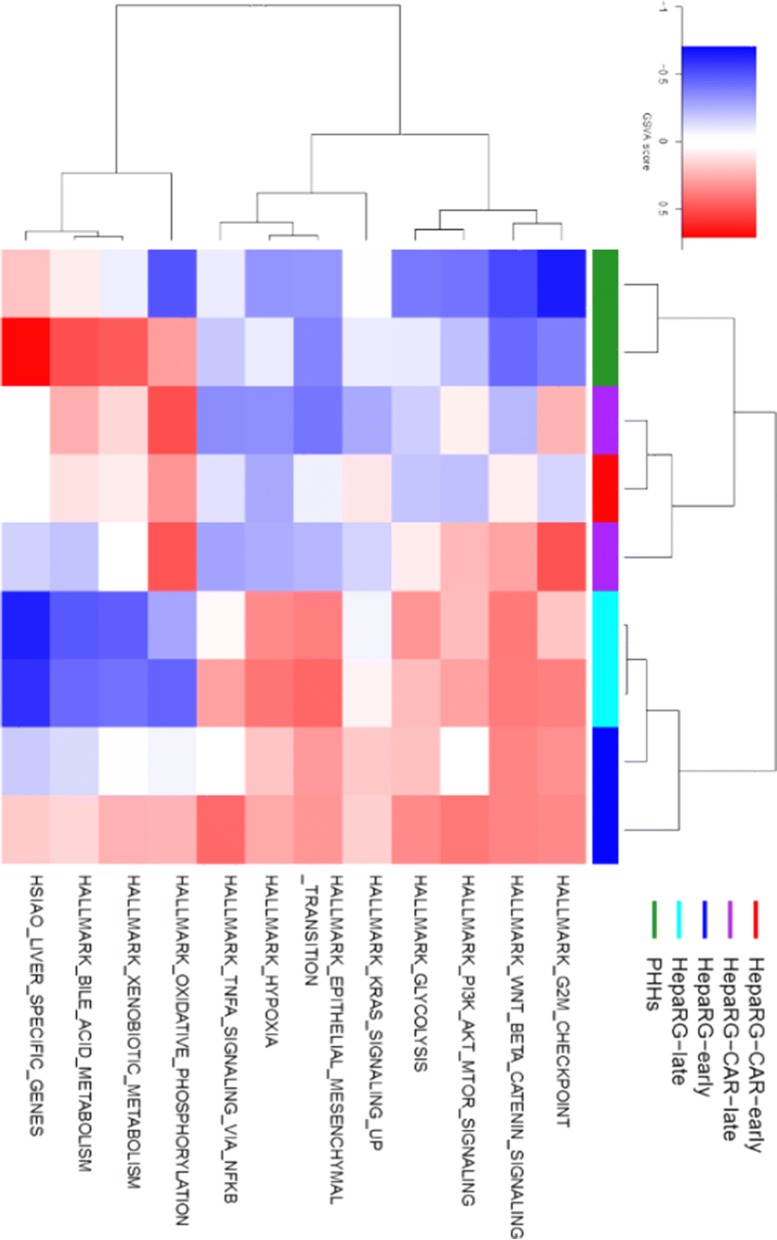
Upon passaging, the transcriptional profile of HepaRG cells shifts towards dedifferentiation and cell proliferation with enhanced glycolysis. Heatmap of the sample-specific geneset enrichment scores determined by CAMERA analysis on selected genesets from top altered (FDR < 0.1) Hallmark genesets and the HSIAO liver-specific geneset of different comparisons. (Color figure online)

Geneset enrichment analysis was employed to identify genesets enriched in DEGs. The Hallmark genesets that include 50 genesets derived from the Molecular Signature Database (MSigDB) (Liberzon et al. 2015), and the HSIAO liver-specific geneset, which contains 238 liver-specific genes (Hsiao et al. 2001), in more detail, were employed to assess different biological processes and hepatic differentiation, resp.. Furthermore, we visualized the relatedness of the different groups for ten selected discriminative genesets involved in energy metabolism, hepatic differentiation and cell proliferation in a heatmap (Fig. 3, for better resolution image, refer to Online Resource 7). These genesets were selected from differentially regulated Hallmark genesets of early- and late-passages from both HepaRG and HepaRG-CAR cells and PHHs. Among the top 19 differentially regulated (FDR < 0.1) genesets, there were 18 upregulated genesets and only one geneset that was downregulated in HepaRG early-passage vs. HepaRG-CAR early-passage, (Table 3, for the complete gene-set analysis, refer to Online Resource 2). Among the upregulated genesets, six genesets were related to cell proliferation and four genesets were involved in inflammatory response. Interestingly, hypoxia and glycolysis Hallmark genesets were upregulated, whereas oxidative phosphorylation (OxPhos) was downregulated in HepaRG early-passage vs. HepaRG-CAR early-passage (Fig. 3, Online Resource 7). This, together with the relatively high lactate production suggests that HepaRG cells highly depend on glycolysis to obtain their energy, whereas HepaRG-CAR cells are more dependent on mitochondrial energy metabolism. Despite the improvement of some hepatic functions (albumin synthesis, bile acid synthesis, lactate metabolism, and, in part, biotransformation) (Catapano et al. 1996), neither the HSIAO liver-specific geneset (FDR = 0.41), nor Hallmark genesets related to hepatic functions were overrepresented in early passages of HepaRG-CAR vs. HepaRG cells.

**Table 3:** Top differentially regulated genesets in HepaRG early-passage vs. HepaRG-CAR early-passage

Gene set	N genes	Direction	FDR
HALLMARK_MYC_TARGETS_V1	197	Up	2.41E−07
HALLMARK_E2F_TARGETS	199	Up	6.73E−06
HALLMARK_HYPOXIA	198	Up	1.74E−05
HALLMARK_G2M_CHECKPOINT	198	Up	2.39E−05
HALLMARK_EPITHELIAL_MESENCHYMAL_TRANSITION	197	Up	7.26E−05
HALLMARK_GLYCOLYSIS	198	Up	1.54E−03
HALLMARK_TNFA_SIGNALING_VIA_NFKB	197	Up	4.08E−03
HALLMARK_MTORC1_SIGNALING	199	Up	7.45E−03
HALLMARK_ALLOGRAFT_REJECTION	187	Up	1.08E−02
HALLMARK_INFLAMMATORY_RESPONSE	193	Up	1.46E−02
HALLMARK_OXIDATIVE_PHOSPHORYLATION	199	Down	2.72E−02
HALLMARK_APOPTOSIS	157	Up	3.10E−02
HALLMARK_UNFOLDED_PROTEIN_RESPONSE	113	Up	3.31E−02
HALLMARK_DNA_REPAIR	147	Up	3.36E−02
HALLMARK_PI3K_AKT_MTOR_SIGNALING	104	Up	4.11E−02
HALLMARK_UV_RESPONSE_DN	143	Up	5.14E-02
HALLMARK_TGF_BETA_SIGNALING	53	Up	5.20E−02
HALLMARK_INTERFERON_GAMMA_RESPONSE	195	Up	6.54E−02
HALLMARK_UV_RESPONSE_UP	153	Up	9.93E−02

For the complete CAMERA analysis of the differential gene-set expression refer to Online Resource 2

Furthermore, to assess the degree of hepatic and metabolic competence of HepaRG cells, we compared the transcriptional profile of HepaRG early-passage to that of PHHs. In the top 20 differentially regulated genesets, 15 and five genesets were up- and downregulated, resp. (Table 4, for the complete gene-set analysis refer to Online Resource 3). Nine of the 15 upregulated genesets were involved in cell proliferation. Other upregulated genesets appearing in the top 20 list were related to unfolded protein response, protein secretion and UV response. The five downregulated genesets were all associated to liver functions, including the HSIAO liver-specific geneset, the xenobiotic detoxification, fatty acid oxidation, bile acid metabolism and coagulation genesets. This outcome indicates the lower hepatic differentiation grade of HepaRG cells compared to PHHs.

**Table 4:** Top 20 differentially regulated genesets in HepaRG early-passage vs. PHHs

Gene set	N genes	Direction	FDR
HSIAO_LIVER_SPECIFIC_GENES	238	Down	1.46E−27
HALLMARK_MYC_TARGETS_V1	197	Up	1.03E−13
HALLMARK_E2F_TARGETS	199	Up	1.68E−13
HALLMARK_G2M_CHECKPOINT	198	Up	1.83E−12
HALLMARK_MITOTIC_SPINDLE	198	Up	3.14E−06
HALLMARK_XENOBIOTIC_METABOLISM	197	Down	5.69E−06
HALLMARK_BILE_ACID_METABOLISM	111	Down	7.27E−06
HALLMARK_DNA_REPAIR	147	Up	4.91E−05
HALLMARK_MYC_TARGETS_V2	58	Up	1.09E−04
HALLMARK_MTORC1_SIGNALING	199	Up	1.43E−04
HALLMARK_P53_PATHWAY	196	Up	1.82E−04
HALLMARK_PROTEIN_SECRETION	96	Up	2.06E−04
HALLMARK_UNFOLDED_PROTEIN_RESPONSE	113	Up	3.64E−04
HALLMARK_WNT_BETA_CATENIN_SIGNALING	40	Up	5.33E−04
HALLMARK_APICAL_JUNCTION	192	Up	5.48E−04
HALLMARK_EPITHELIAL_MESENCHYMAL_TRANSITION	197	Up	6.09E−04
HALLMARK_COAGULATION	131	Down	8.11E−03
HALLMARK_GLYCOLYSIS	198	Up	9.47E−03
HALLMARK_UV_RESPONSE_DN	143	Up	1.10E−02
HALLMARK_FATTY_ACID_METABOLISM	156	Down	1.15E−02

For the complete CAMERA analysis of the differential gene-set expression refer to Online Resource 3

The top 20 differentially regulated genesets in HepaRG-CAR early-passage vs. PHHs was highly similar to that of the HepaRG early-passage vs. PHH comparison with 15 upregulated genesets including nine related to cell proliferation and the same five downregulated genesets (Table 4). In line with the observation that HepaRG-CAR cells show relatively high lactate metabolism, the OxPhos was more induced in HepaRG-CAR early-passage than in PHHs (Table 5-for the complete gene-set analysis refer to Online Resource 4 and Fig. 3 and Online Resource 7), however, it should be noted that PHHs replicates displayed a variable OxPhos profile, which complicates the interpretation of the heatmap (Fig. 3, Online Resource 7). In brief, these data show that the hepatic differentiation of HepaRG-CAR cells is also still underdeveloped when compared to PHHs.

**Table 5:** Top 20 differentially regulated genesets in HepaRG-CAR early-passage vs. PHHs

Gene set	N genes	Direction	FDR
HSIAO_LIVER_SPECIFIC_GENES	238	Down	3.87E−28
HALLMARK_G2M_CHECKPOINT	198	Up	5.40E−10
HALLMARK_E2F_TARGETS	199	Up	6.56E−10
HALLMARK_MYC_TARGETS_V1	197	Up	1.76E−08
HALLMARK_XENOBIOTIC_METABOLISM	197	Down	5.79E−06
HALLMARK_MITOTIC_SPINDLE	198	Up	1.15E−05
HALLMARK_BILE_ACID_METABOLISM	111	Down	2.91E−05
HALLMARK_P53_PATHWAY	196	Up	1.45E−03
HALLMARK_DNA_REPAIR	147	Up	1.56E−03
HALLMARK_WNT_BETA_CATENIN_SIGNALING	40	Up	1.84E−03
HALLMARK_MYC_TARGETS_V2	58	Up	1.87E−03
HALLMARK_COAGULATION	131	Down	3.28E−03
HALLMARK_PROTEIN_SECRETION	96	Up	3.50E−03
HALLMARK_APICAL_JUNCTION	192	Up	4.81E−03
HALLMARK_MTORC1_SIGNALING	199	Up	8.29E−03
HALLMARK_FATTY_ACID_METABOLISM	156	Down	1.12E−02
HALLMARK_UNFOLDED_PROTEIN_RESPONSE	113	Up	1.51E−02
HALLMARK_EPITHELIAL_MESENCHYMAL_TRANSITION	197	Up	1.63E−02
HALLMARK_ANGIOGENESIS	35	Up	1.83E−02
HALLMARK_OXIDATIVE_PHOSPHORYLATION	199	Up	5.15E−02

For the complete CAMERA analysis of the differential gene-set expression refer to Online Resource 4

To study the effect of passaging on the transcriptional profile of HepaRG and HepaRG-CAR cells, we compared the transcriptional profile of the Hallmark genesets and HSIAO liver specific-geneset of early-passage to that of late-passage within the same cell line.

In HepaRG late-passage vs. HepaRG early-passage, only 16 differentially regulated genesets, with FDR < 0.1, were identified, including five upregulated and 11 downregulated genesets (Table 6, for the complete gene-set analysis refer to Online Resource 5). Three of the upregulated Hallmark genesets were linked to cell cycle and proliferation (3/5), in agreement with the trend of increased protein synthesis at late passages. The same liver function-associated genesets differentially regulated between early passages of HepaRG and HepaRG-CAR vs. PHHs were downregulated in the top differentially regulated genesets of HepaRG late- vs. HepaRG early-passage, indicating loss of hepatic differentiation upon passaging of HepaRG cells, which was further confirmed by upregulation of the epithelial-mesenchymal transition geneset. In line with increased lactate production upon passaging, the bioenergetics profile of HepaRG cells was shifted to suppression of OxPhos and induction of hypoxia related genesets. The shift in energy metabolism by decreasing the OxPhos activity was further reflected by the downregulation of the geneset related to reactive oxygen species (ROS), (Fig. 3, Online Resource 7). Furthermore, two immunity-related, interferon-response genesets were downregulated upon passaging of HepaRG.

**Table 6:** Top differentially regulated genesets (16 genesets) in HepaRG late-passage vs. HepaRG early-passage

Gene set	N genes	Direction	FDR
HSIAO_LIVER_SPECIFIC_GENES	238	Down	1.52E−22
HALLMARK_XENOBIOTIC_METABOLISM	197	Down	1.63E−08
HALLMARK_FATTY_ACID_METABOLISM	156	Down	3.72E−07
HALLMARK_BILE_ACID_METABOLISM	111	Down	3.12E−06
HALLMARK_INTERFERON_ALPHA_RESPONSE	96	Down	3.82E−06
HALLMARK_INTERFERON_GAMMA_RESPONSE	195	Down	2.12E−05
HALLMARK_HYPOXIA	198	Up	2.38E−05
HALLMARK_OXIDATIVE_PHOSPHORYLATION	199	Down	4.48E-05
HALLMARK_ADIPOGENESIS	194	Down	1.49E−03
HALLMARK_EPITHELIAL_MESENCHYMAL_TRANSITION	197	Up	8.09E−03
HALLMARK_MYC_TARGETS_V1	197	Up	1.25E−02
HALLMARK_REACTIVE_OXIGEN_SPECIES_PATHWAY	47	Down	1.70E−02
HALLMARK_COAGULATION	131	Down	1.73E−02
HALLMARK_PEROXISOME	101	Down	1.80E−02
HALLMARK_DNA_REPAIR	147	Up	2.08E−02
HALLMARK_P53_PATHWAY	196	Up	3.43E−02

For the complete CAMERA analysis of the differential gene-set expression refer to Online Resource 5

There were only eight differentially regulated genesets in HepaRG-CAR late-passage vs. HepaRG-CAR early-passage of which six were upregulated and two downregulated (Table 7, for the complete gene-set analysis refer to Online Resource 6). All of the six upregulated genesets were related to cell proliferation, compatible with the increased protein synthesis at later passage. Interestingly, upon passaging of HepaRG-CAR cells the Hallmark geneset epithelial mesenchymal transition was downregulated, and there was no effect on hepatic differentiation as the transcriptional profile of the HSIAO liver-specific-geneset was not significantly altered (FDR = 0.43), and none of the genesets associated to hepatic functions appeared in the top differentially regulated genesets. In addition, the angiogenesis process, which is critically important for cancer cells to meet their increasing demand for nutrient supply (Folkman 1971; Nishida 2005; Nishida et al. 2006), was downregulated upon the passaging of HepaRG-CAR cells. These results are pointing to a more stable hepatic and energy metabolic profile of HepaRG-CAR cells upon passaging compared to HepaRG cells, in line with the functional data.

**Table 7:** Top differentially regulated genesets (8 genesets) in HepaRG-CAR late-passage vs. HepaRG-CAR early-passage

Gene set	N genes	Direction	FDR
HALLMARK_MYC_TARGETS_V1	197	Up	1.02E−08
HALLMARK_E2F_TARGETS	199	Up	1.20E−08
HALLMARK_G2M_CHECKPOINT	198	Up	1.52E−05
HALLMARK_DNA_REPAIR	147	Up	1.54E−05
HALLMARK_MYC_TARGETS_V2	58	Up	3.45E−03
HALLMARK_EPITHELIAL_MESENCHYMAL_TRANSITION	197	Down	6.31E−02
HALLMARK_ANGIOGENESIS	35	Down	9.23E−02
HALLMARK_MTORC1_SIGNALING	199	Up	9.55E−02

For the complete CAMERA analysis of the differential gene-set expression refer to Online Resource 6

The heatmap (Fig. 3, Online Resource 7), confirms the general picture of a sustained stability of HepaRG-CAR cells upon passaging, in contrast to the parental HepaRG cells, where passaging exerted alterations in favor of loss of differentiation and epithelial-mesenchymal transition. In agreement with the PCA analysis (Fig. 2), the heatmap showed a clear clustering of the HepaRG-CAR cells (regardless of passaging) and the HepaRG early-passage, whereas the HepaRG late-passage cells clustered separately. Furthermore, CAR overexpression shifted the transcriptional profile of HepaRG cells from glycolysis towards mitochondrial energy metabolism, however, the effect on hepatic differentiation at transcript level seems low, resulting in a considerable gap in hepatic differentiation between HepaRG ± CAR cells and PHHs.

### In contrast to HepaRG, HepaRG-CAR cells produce less ROS

Oxidative stress is a driving force in aging processes, as well as diverse liver pathologies (Davalli et al. 2016; Kong et al. 2014). Oxidative stress reflects the balance between the generation of ROS, as byproduct of aerobic energy metabolism and the antioxidant system which detoxifies these reactive molecules (Lee et al. 2012; Li et al. 2015; Godoy et al. 2015). Important enzymes involved in ROS detoxification include superoxide dismutase (SOD) 1 and 2 and catalase (CAT) (Ghosh et al. 2013; Jones et al. 1993; Sea et al. 2015).

**Fig. 4:**
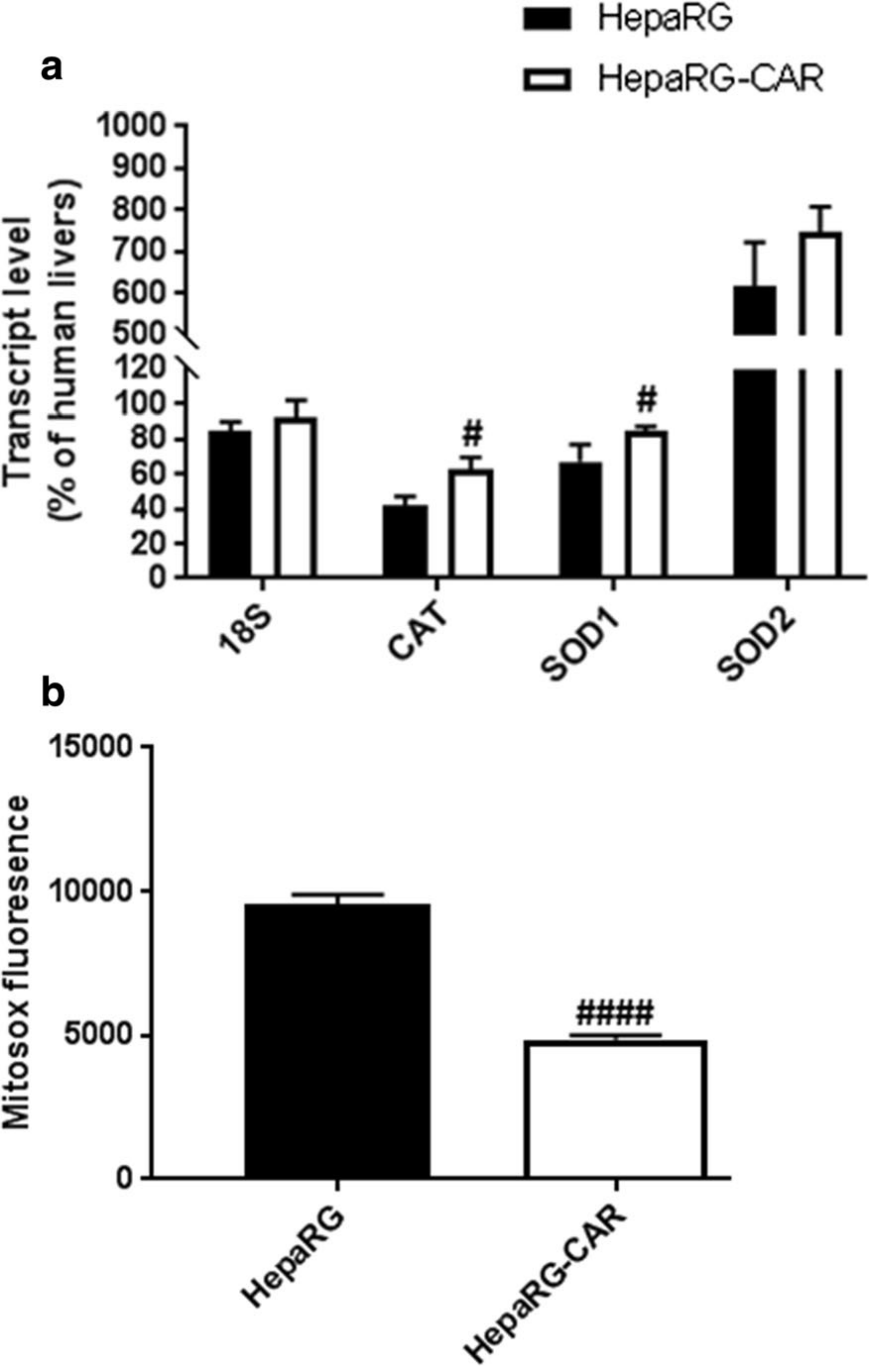
HepaRG-CAR cells produce less ROS. **a** The transcript levels of antioxidant genes expressed as a % of human livers. Also shown is the level of *18S* ribosomal RNA, which was not changed in HepaRG-CAR line vs. HepaRG cells and was comparable to human liver level, and was used as reference gene for normalization of the RT-qPCR data (Bustin et al. 2009). **b** ROS production (n = 4/2 exp). Data was represented as mean ± SD; *P*-value < 0.05 was considered as significant

We tested whether the difference in stability between HepaRG and HepaRG-CAR may be associated with oxidative stress changes. Interestingly, the transcript levels of *CAT* and *SOD1* were significantly higher in HepaRG-CAR vs. HepaRG cells at early-passage, whereas *SOD2* showed a positive trend (Fig. 4a). Furthermore, mitochondrial superoxide level was 2.0-fold lower in HepaRG-CAR vs. HepaRG cells (Fig. 4b), indicating that HepaRG-CAR cells are less exposed to ROS than HepaRG cells.

## Discussion

There is an increasing demand for terminally-differentiated hepatocyte cultures to function as a hepatocyte-based in vitro model for human liver as well as biocomponent of BALs. The most functional proliferative source of human hepatocytes to date is the human liver cell line HepaRG, which shows a relatively broad spectrum of liver functions (Gripon et al. 2002; Hoekstra et al. 2011). In this study we demonstrate that, compared to the parental HepaRG cell line, the newly developed HepaRG-CAR cell line displays higher mitochondrial function, and, in part, higher hepatic differentiation, as well as increased stability upon passaging, and therefore outperforms the parental HepaRG cell line as proliferative source of human hepatocytes.

Previously it was established that HepaRG-CAR cells showed increased biotransformation, particularly at activity level, and albumin production compared to HepaRG cells (van der Mark et al. 2017). The current study additionally shows increased mitochondrial function, as reflected by lower lactate production, and also a higher bile acid production. Accordingly, the RNA-seq expression analysis confirmed the induction of OxPhos in HepaRG-CAR vs. HepaRG cells whereas glycolysis, hypoxia and proliferation processes were downregulated.

CAR is implicated not only in biotransformation through regulating the transcript level of a large array of genes involved in biotransformation, however, it also is involved, among others, in bile acid synthesis and in energy homeostasis by governing lipogenesis and gluconeogenesis (Bechmann et al. 2012; Chiang 2009; Cui and Klaassen 2016). The upregulation of bile acid synthesis is in line with our previous findings that several CYP enzymes are induced at transcript and/or activity level in HepaRG-CAR cells (van der Mark et al. 2017), as CYP enzymes are involved in the synthesis of bile acids from cholesterol (Cui and Klaassen 2016; Petrick and Klaassen 2007; Blazquez et al. 2013).

Several other studies suggested CAR as a critical transcription factor for regulating hepatocyte differentiation. CAR forms a transcriptional regulatory network with other nuclear receptors that are involved in hepatic differentiation, as hepatic nuclear factors 1 and 4 (Chen et al. 2018). Chen et al. demonstrated that lentiviral CAR-overexpression promotes the differentiation and maturation of human embryonic stem cells into hepatocyte-like cells (Chen et al. 2013). CAR further stimulates hepatic differentiation indirectly by promoting the synthesis of bile acids which, on their turn, activate the farnesoid X receptor and small heterodimer partner (FXR/SHP) signaling axis (Chiang 2013, 2017; Hoeke et al. 2014). Godoy et al. indicated FXR, CAR, pregnane X receptor and hepatic nuclear factor 1 as key transcription factors of a cluster of hepatic genes with low, deregulated, expression levels in stem cell-derived hepatocyte like cells and dedifferentiated PHHs relative to fully differentiated PHHs (Godoy et al. 2015, 2016). The genes overrepresented in this cluster were involved in biotransformation processes, most often CYPs (Godoy et al. 2015). These findings were confirmed by proteomic analyses showing that PHH dedifferentiation is particularly associated with changes in CYP levels (Heslop et al. 2017). Interestingly, the other group of proteins associated with PHH dedifferentiation comprised of mitochondrial proteins. This is in agreement with a large-scale transcriptomic and proteomic study, which reported that early changes associated with hepatic dedifferentiation related, in part, to inhibition of major metabolic pathways taking place in the mitochondria, such as TCA cycle, β-oxidation of fatty acids and oxidative phosphorylation (Lauschke et al. 2016). Both CYPs and energy metabolism are, at least partly, under transcriptional control of CAR, therefore it is likely that CAR upregulation will inhibit the dedifferentiation of PHHs.

The mechanistic processes downstream of CAR in regulating energy homeostasis are still under investigation. These studies are complicated by species-specific differences between rodent and human CAR for their gene targets and mode of activity (Niu et al. 2018; Yang et al. 2014). Highly intriguing, however, is the new finding that the signaling of the mammalian target of rapamycin complex 1 (mTORC1) is downregulated in HepaRG-CAR vs. HepaRG cells, as well as the Myc target genesets (Table 2). The mTORC1 complex is a nutrient sensor that, through activation of c-myc, drives cancer progression and metabolic reprogramming, characterized by upregulation of glycolysis, despite the presence of sufficient oxygen, and simultaneously limiting OxPhos, a phenomenon known as the Warburg effect (Harachi et al. 2018; Vander Heiden et al. 2009; Warburg 1956). In line with this, our study shows that CAR overexpression is associated with the reversion of the Warburg effect in HepaRG cells, with downregulation of glycolysis and upregulation of OxPhos genesets, which may be mediated by the mTORC1 pathway through c-myc. Interestingly, Parent et al. showed that sustained mTOR activity induced a preneoplastic phenotype to HepaRG cells by altering the translation of genes vital for establishing normal hepatic energy homeostasis and moderating hepatocellular growth (Parent et al. 2007). Moreover, these neoplastic changes were reversible upon administration of the classic mTORC1 inhibitor rapamycin.

In parallel, mTORC1 signalling and c-myc, being well-known for their tumor-promoting effects, may play an important role in the transformation of HepaRG cells normally occurring at P20, and characterized by morphological changes, decreased ammonia elimination and bile acid synthesis and increased lactate production, which is inhibited in HepaRG-CAR cells. Changes in expression profile associated with the transformation in HepaRG cells included downregulation of genesets associated with hepatic functions and induction of epithelial mesenchymal transition and hypoxia. Moreover, several cell proliferation related genesets were induced, further confirming the epithelial mesenchymal transition. These manifestations, also described in (Heslop et al. 2017; Niu et al. 2018), as associated with the dedifferentiation of PHHs in culture, are characteristic for the Warburg effect (Vander Heiden et al. 2009; Warburg 1956).

Thus far, there are no studies about the mechanism governing the dedifferentiation process in HepaRG cells. However, several studies demonstrated the involvement of oxidative stress in pathological mechanisms of a vast range of liver pathologies (Li et al. 2015), as well as mitochondrial dysfunction and the Warburg effect (Vander Heiden et al. 2009; Warburg 1956). Oxidative stress is caused by high levels of ROS, the net result of production through oxidative phosphorylation and scavenging by antioxidant activity. The production of ROS may be increased in HepaRG-CAR cells, due to its increased mitochondrial energy metabolism. However, and possibly due to the induction of antioxidant enzymes, the net amount of ROS was reduced to 50% of the level of HepaRG cells. Given the pronounced effect on ROS production and the relatively limited upregulation of *SOD1* and *CAT* mRNA, posttranslational modifications of these antioxidant enzymes or other antioxidant systems may also be involved (Cao et al. 2003; Glorieux et al. 2015; Miao and St Clair 2009). The relatively low ROS levels may, at least, partly attribute to HepaRG-CAR cells long-term stability. This finding seems in conflict with the downregulation of the ROS-related geneset in HepaRG cells late vs. early-passage, which was accompanied by the drastic loss of mitochondrial energy metabolism as shown by the suppression of genesets related to OxPhos and fatty acid metabolism and the increased production of lactate. The level and role of ROS may be dynamically regulated during passaging and may differ for both cell lines, which needs to be further analyzed. In addition, the role of mTORC1 signaling should be further analyzed. mTORC1 may play an essential role in the transformation of HepaRG cells, as inhibition of mTORC1 has been shown to delay age-related diseases, due to stimulation of autophagy and possibly modulation of immune responses (Meijer and Codogno 2007; Weichhart 2018).

The transcriptional profile of HepaRG cells early-passage was dissimilar from that of the PHHs, in line with our previous study (Adam et al. 2018b). The transcriptional profile of HepaRG-CAR cells, regardless of the passage number, clustered closely with HepaRG cells at early-passage and differed from that of PHHs. Interestingly, the cellular bioenergetics of HepaRG-CAR cells (regardless of passage number) was shifted towards the PHHs profile with the induction of OxPhos and inhibition of hypoxia and glycolysis Hallmark genesets. Yet, when related to PHHs, xenobiotic detoxification, bile acid metabolism and HSIAO liver specific-geneset were downregulated in HepaRG-CAR cells, indicating that there is still room for further improvement to advance their hepatic differentiation. However, the relatively high heterogeneity in the transcriptional profile of the PHH replicates, complicates the interpretation of the transcriptional data in relation to PHHs. This may be, in part, related to the optional dedifferentiation of the PHHs that involves down-regulation of important liver-enriched transcription factors and subsequently loss of hepatic differentiation (Elaut et al. 2006). It is widely believed that the dedifferentiation process is initiated, as early as, during hepatocyte isolation phase and it progressively continues during their in vitro cultivation (Elaut et al. 2006; Godoy et al. 2009; Zellmer et al. 2010). To limit misinterpretation of transcriptional data, due to this optional PHH dedifferentiation, we used isolated PHHs without in vitro cultivation as reference materials. The PCA analysis of the full transcriptome showed that the differences between the two PHH isolates were relatively small compared to differences between cell lines and differences within HepaRG cultures over time. However, the PHH replicates displayed substantial variations in their transcriptional profile for specific genesets, particularly for those related to OxPhos and liver-related functions, which may be related to biological variation between patients. The observed upregulation of hepatic functions, including albumin and bile acid synthesis, as well as a range of biotransformation activities in HepaRG-CAR cells vs. HepaRG cells (van der Mark et al. 2017), seems in conflict with the lack of upregulated genesets associated with hepatic differentiation in the transcriptome analysis. This suggests that the effects of CAR overexpression most probably are mediated at posttranscriptional level. The shift in energy metabolism by CAR overexpression may modulate hepatic functions due to alteration in metabolite spectrum that governs the flux rates of many metabolic pathways (Madiraju et al. 2016) and to increased fluxes of energy-consuming processes, such as biotransformation. Moreover, the accumulation of metabolites normally eliminated by the mitochondria, such as lactate, will be inhibited, which may improve hepatic functionality (Catapano et al. 1996). Furthermore, CAR overexpression enhances the mRNA expression of CYP450 oxidoreductase (*POR*) (van der Mark et al. 2017), which mediates the electron transfer required for P450 activity (Gutierrez et al. 2003). Therefore, the upregulation of POR may contribute at posttranscriptional level to the increased biotransformation activity of HepaRG-CAR. It will be, however, difficult to distinguish between the different players that may post-transcriptionally increase the hepatic functionality of HepaRG cells upon CAR overexpression, due to interconnections between the processes regulated by the concentration of metabolites and energy derivatives.

## Conclusions

Given the advanced biotransformation properties, mitochondrial functions and the sustained stability, this work demonstrates that, the newly-developed HepaRG-CAR cell line is more suitable as an attractive alternative to PHHs for BAL application and for in vitro drug toxicity studies. For the support of patients with end-stage liver failure it is estimated that a functional liver mass of 150 g (15 × 10^9^ cells) is required (Tsiaoussis et al. 2001). This implies that at least 11 passages from the first single HepaRG cell are needed to load a single BAL. Therefore, the extended stability of the HepaRG-CAR cells upon passaging is essential for BAL application. Other applications requiring human liver cells, as pre-clinical studies in drug development and research on liver functions and infections, require substantial less cells, however, also for those applications, the highest hepatic functionality is required, and therefore also in those cases the HepaRG-CAR cells are the best available alternative to PHHs.

## Electronic supplementary material

Below is the link to the electronic supplementary material.

Supplementary material 1 (xlsx 19 kb) Excel file with functionality and mRNA data.

Supplementary material 2 (txt 1,406 kb) The complete CAMERA analysis of the differential geneset expression in HepaRG early-passage vs. HepaRG-CAR early-passage.

Supplementary material 3 (txt 1,428 kb) The complete CAMERA analysis of the differential geneset expression in HepaRG early-passage vs. PHHs.

Supplementary material 4 (txt 1,420 kb) The complete CAMERA analysis of the differential geneset expression in HepaRG-CAR early-passage vs. PHHs.

Supplementary material 5 (txt 1,408 kb) The complete CAMERA analysis of the differential geneset expression in HepaRG late-passage vs. HepaRG early-passage.

Supplementary material 6 (txt 1,411 kb) The complete CAMERA analysis of the differential geneset expression in HepaRG-CAR late-passage vs. HepaRG-CAR early-passage.

Supplementary material 7 (tif 169 kb) High resolution image for the Heatmap of the sample-specific geneset enrichment scores determined by CAMERA analysis on selected genesets from top altered (FDR < 0.1) Hallmark genesets and the HSIAO liver-specific geneset of different comparisons.
