# Supplementary figures and images for: Genome-wide expression profiling reveals increased stability and mitochondrial energy metabolism of the human liver cell line HepaRG-CAR
Source: Cytotechnology. 2020 Mar 4;72(3):377–95. doi: 10.1007/s10616-020-00384-w (PMC7225227; doi:10.1007/s10616-020-00384-w)

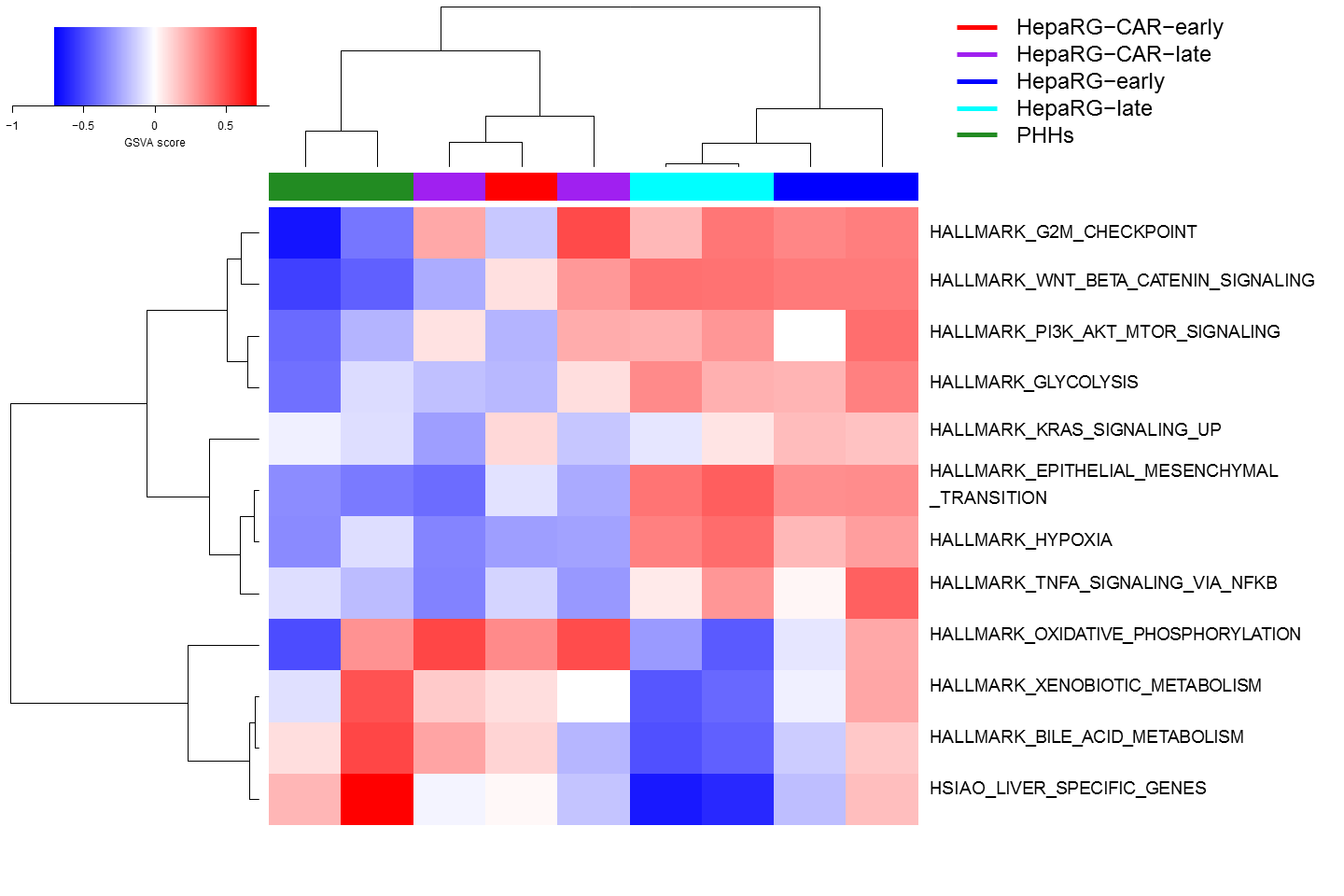

Supplement: Supplementary file 7 — Supplementary material 7 (tif 169 kb) High resolution image for the Heatmap of the sample-specific geneset enrichment scores determined by CAMERA analysis on selected genesets from top altered (FDR < 0.1) Hallmark genesets and the HSIAO liver-specific geneset of different comparisons. [file 10616_2020_384_MOESM7_ESM.tif]
